# Association Between Preoperative C-reactive Protein and Early Postoperative Complications in Patients With Colorectal Cancer: A Prospective Observational Study

**DOI:** 10.7759/cureus.117856

**Published:** 2026-10-13

**Authors:** Md. Ashraful Islam, Md. Tarek Hasan, Saoda Islam, Munsur Miah, Muhammad Ali Siddiquee, Tamanna Islam Lima, Mahmud Hasan

**Affiliations:** 1 Department of Surgery, Dhaka Medical College and Hospital, Dhaka, BGD; 2 Department of Surgery, Directorate General of Health Services, Dhaka, BGD; 3 Department of Orthopedics, Sher-e-Bangla Medical College, Barishal, BGD; 4 Department of Colorectal Surgery, Bangladesh Medical University, Dhaka, BGD; 5 Department of Surgery, Shaheed Tajuddin Ahmad Medical College, Gazipur, BGD

**Keywords:** clavien-dindo complications classification, colorectal cancer, c-reactive protein, postoperative complications, predictive biomarker

## Abstract

Background

Postoperative complications are important adverse outcomes after colorectal cancer (CRC) surgery. Preoperative C-reactive protein (CRP), an accessible marker of systemic inflammation, may provide useful information about the risk of postoperative morbidity. This study aimed to evaluate the association between preoperative CRP and postoperative complications in patients undergoing elective CRC surgery and to assess its ability to discriminate complication severity.

Methods

This prospective observational study included 64 patients undergoing elective, potentially curative CRC surgery at the Department of Colorectal Surgery, Bangladesh Medical University, Dhaka, between March 2024 and February 2025. Preoperative CRP was measured, with concentrations >5 mg/L considered elevated. Postoperative complications occurring during hospitalization were recorded and graded according to the Clavien-Dindo classification. Associations between CRP and postoperative complications were assessed using the chi-square test or Fisher's exact test, as appropriate.

Results

Among the 64 patients included, 42 (65.6%) were male patients. The mean age was 46.6 ± 15.3 years. Preoperative CRP was >5 mg/L in 24 (37.5%) patients. Overall, postoperative complications occurred in 43 (67.2%) patients, including wound infection in 17 (26.6%), anastomotic leakage in 6 (9.4%), respiratory complications in 6 (9.4%), and renal complications in 1 (1.6%). Patients with CRP >5 mg/L had significantly higher rates of wound infection (58.3% vs. 7.5%; P<0.001), respiratory complications (20.8% vs. 2.5%; P=0.024), and anastomotic leakage (25.0% vs. 0%; P=0.002) than those with CRP ≤5 mg/L. Among the 43 patients who developed postoperative complications, 10 (23.3%) had Clavien-Dindo grade ≥III complications. All patients with grade ≥III complications had CRP >5 mg/L, compared with 12 (36.4%) patients with grade I-II complications (P<0.001).

Conclusion

Elevated preoperative CRP was associated with postoperative morbidity and greater complication severity in this cohort. Preoperative CRP may serve as an adjunct to perioperative risk assessment, although larger studies with independent validation are needed.

## Introduction

Colorectal cancer (CRC) is a major contributor to the global cancer burden and remains an important cause of cancer-related morbidity and mortality [1]. Surgical resection offers the primary opportunity for curative treatment in patients with localized and locally advanced disease. Despite improvements in surgical techniques and perioperative care, complications after colorectal surgery remain relatively common and can substantially affect postoperative recovery.

Postoperative morbidity following colorectal resection encompasses a wide range of events, including surgical-site infection, anastomotic leakage, intra-abdominal infection, pulmonary complications, renal dysfunction, and other systemic complications [2-5]. These adverse events may lead to prolonged hospitalization, greater healthcare utilization, postponement of adjuvant treatment, and deterioration in overall clinical outcomes. Consequently, identification of readily available preoperative markers that can help recognize patients at increased risk of postoperative complications is of practical clinical interest.

Inflammation is increasingly recognized as an important component of CRC pathophysiology. C-reactive protein (CRP) is a commonly measured acute-phase reactant whose circulating concentration increases in response to systemic inflammatory signaling, particularly through interleukin-6-mediated pathways. Because CRP testing is inexpensive, widely available, and technically straightforward, it represents a potentially useful marker for assessing systemic inflammatory activity in patients with CRC [6-9].

Several studies have explored the relationship between preoperative inflammatory status and postoperative outcomes. Elevated inflammatory markers before colorectal cancer surgery have been associated with an increased occurrence of postoperative infectious complications [6]. Similarly, higher preoperative CRP concentrations have been reported in association with postoperative morbidity following colorectal surgery [7]. These observations provide a rationale for examining whether CRP measured before surgery can identify patients who are more likely to experience complications during the early postoperative period.

Preoperative CRP has also attracted attention as a prognostic biomarker in CRC. Previous meta-analytic studies have reported associations between increased CRP levels before surgery and unfavorable survival outcomes [8]. Other investigations have suggested that CRP may provide supplementary prognostic information in patients undergoing colorectal cancer resection with curative intent [9]. However, prognostic utility and prediction of short-term postoperative morbidity represent different clinical applications and should not be considered interchangeable.

Evidence regarding the ability of preoperative CRP to predict specific postoperative complications is also inconsistent. A systematic review and meta-analysis comprising 23 studies and 7,147 patients reported that elevated preoperative CRP was associated with postoperative infectious complications. In contrast, significant associations were not demonstrated when anastomotic leakage was assessed separately or when overall postoperative morbidity was considered as the outcome [10]. Such findings suggest that the relationship between preoperative inflammation and postoperative complications may vary according to the definition and severity of the postoperative outcome being evaluated.

Most available evidence has been generated in healthcare systems with greater resources and more established perioperative infrastructure. Comparable data from Bangladesh are limited. In resource-constrained settings, the clinical utility of routinely available and relatively inexpensive biomarkers may be particularly relevant because they can potentially support risk assessment without requiring specialized investigations.

Therefore, this prospective observational study evaluated the association between preoperative CRP and early postoperative complications in patients undergoing elective CRC surgery at Bangladesh Medical University (BMU). The study specifically examined whether an elevated preoperative CRP level was associated with the occurrence and severity of postoperative complications.

## Materials and methods

Study design and setting

This prospective observational study was conducted in the Department of Colorectal Surgery, BMU, Dhaka, Bangladesh, from March 2024 to February 2025. Adult patients with histologically confirmed colorectal carcinoma who underwent elective potentially curative colorectal cancer surgery were prospectively enrolled.

The primary outcome was the occurrence of at least one postoperative complication during the index hospitalization, classified according to the Clavien-Dindo system. The study evaluated the association between preoperative CRP levels and the occurrence and severity of postoperative complications.

Inclusion and exclusion criteria

Patients aged >18 years, with histologically confirmed colorectal carcinoma requiring elective or potentially curative resection, patients of either sex, and who provided written informed consent before enrollment were included. Patients were excluded if they required emergency surgery, underwent palliative surgery, or had a documented active infection requiring treatment at the time of preoperative assessment.

Participants and sampling

Eligible patients were recruited using consecutive non-probability sampling. During the study period, consecutive patients who met the predefined eligibility criteria and were available to the study team were approached for participation until the required sample size was reached. Patients who declined participation were not enrolled.

Sample size

The sample size was calculated based on the anticipated sensitivity of preoperative CRP for predicting postoperative complications. Based on a previous study, the expected sensitivity was 55.6%, with an absolute precision of 0.122 (12.2 percentage points) [11]. The required sample size was calculated using the formula for estimation of a single proportion:

$n=\frac{Z^2pq}{d^2}$,

where n is the required sample size, Z is the standard normal deviate corresponding to a 95% confidence level (1.96), p is the anticipated sensitivity of preoperative CRP (0.556), and d is the acceptable absolute precision (0.122).

Substituting the values,

$n=\frac{(1.96)^2(0.556)(0.444)}{(0.122)^2}$

*n≈64.0*.

The calculated sample size was therefore rounded up to 64 participants. Accordingly, a total of 64 patients were enrolled in the study.

Clavien-Dindo classification of postoperative complications

Postoperative complications were classified according to the Clavien-Dindo classification based on the treatment required and the clinical consequences of each complication [5]. Grade I was defined as any deviation from the normal postoperative course that did not require pharmacological treatment or surgical, endoscopic, or radiological intervention. Grade II complications were those requiring pharmacological treatment beyond that permitted for grade I complications, including treatment with antibiotics, blood transfusion, or total parenteral nutrition. Grade III complications were those requiring surgical, endoscopic, or radiological intervention; grade IIIa referred to interventions performed without general anesthesia, whereas grade IIIb referred to interventions requiring general anesthesia. Grade IV complications were life-threatening complications requiring intensive care management, with grade IVa representing single-organ dysfunction and grade IVb representing multiorgan dysfunction. Grade V indicated death of the patient.

For statistical analysis, postoperative complications were categorized into two groups: Clavien-Dindo grade <III, comprising grades I and II, and grade ≥III, representing major postoperative complications. Grade ≥III complications were considered major because they required surgical, endoscopic, or radiological intervention (grade III) or involved life-threatening organ dysfunction, with or without single- or multiorgan failure (grade IV).

Definition of postoperative complications

Postoperative complications were assessed prospectively from the date of surgery until hospital discharge. The predefined observation window therefore corresponded to the index hospitalization, rather than a fixed number of postoperative days.

Wound infection was defined as a superficial or deep surgical-site infection involving the operative wound, based on clinical assessment and, when indicated, microbiological or other diagnostic findings.

Anastomotic leakage was defined as clinically or radiologically confirmed disruption of an intestinal anastomosis resulting in leakage of luminal contents or requiring therapeutic intervention. The number of patients who underwent an intestinal anastomosis was recorded separately, and the anastomotic leakage rate was calculated using the number of patients with an anastomosis as the appropriate denominator.

Respiratory complications were defined as clinically diagnosed pneumonia or respiratory failure requiring additional medical or respiratory support.

Renal complications were defined as acute kidney injury occurring during the postoperative hospitalization. Cardiac complications included new, clinically significant arrhythmia, myocardial infarction, or acute decompensated heart failure.

Each postoperative complication was recorded individually. When a patient experienced more than one complication, the highest Clavien-Dindo grade was used to classify overall postoperative complication severity.

Preoperative CRP measurement

A single preoperative venous blood sample was obtained from each participant during the routine preoperative assessment before colorectal cancer surgery. C-reactive protein was measured using a quantitative immunoturbidimetric assay on a Cobas c 311 automated clinical chemistry analyzer (Roche Diagnostics, Mannheim, Germany) in the Department of Laboratory Medicine, BMU. All CRP measurements were performed in the same laboratory using the same analytical platform and standardized laboratory procedures throughout the study period.

Blood samples were collected within 24 hours before surgery, before any surgical intervention. The CRP measurement therefore represented the patient's preoperative inflammatory status immediately before the planned operation. No repeated preoperative CRP measurements were routinely performed, and the analysis was based on a single CRP measurement for each participant.

A CRP concentration of >5 mg/L was predefined as elevated for the primary categorical analysis, whereas a CRP concentration of ≤5 mg/L was considered within the reference range.

Other preoperative laboratory variables

Preoperative laboratory investigations included complete blood count, erythrocyte sedimentation rate (ESR), and CRP. Hemoglobin, white blood cell (WBC) count, platelet count, and ESR were recorded from investigations performed as part of the routine preoperative assessment. ESR was measured using an automated ESR analyser and reported in millimetres per hour (mm/h). An ESR value >35 mm/h was considered elevated according to the predefined study criterion.

Clinical, tumor, and operative variables

Demographic and clinical variables recorded prospectively included age, sex, body mass index (BMI), smoking status, alcohol consumption, relevant comorbidities, and duration of presenting symptoms.

Tumor-related variables included tumor location, histological diagnosis, tumor grade, and pathological stage. Operative variables included the type of colorectal resection, surgical approach, operative findings, and whether an intestinal anastomosis was performed.

The decision to perform laparoscopic or open surgery was made by the operating surgical team according to routine institutional practice and was based on the patient's clinical condition, tumor characteristics and location, disease extent, previous abdominal surgery where relevant, technical feasibility, and surgeon judgment. No single predefined CRP-based criterion was used to determine the operative approach.

Because the study was observational, the choice of surgical approach was not randomized. Surgical approach and other operative characteristics were therefore considered potential sources of confounding when interpreting the association between preoperative CRP and postoperative complications.

Postoperative follow-up

Patients were followed up prospectively from the time of surgery until discharge from the index hospitalization. The duration of postoperative hospitalization was recorded for each patient. Because follow-up ended at hospital discharge, complications occurring after discharge were not systematically assessed. Consequently, complications such as delayed anastomotic leakage, wound infection, readmission, or other morbidity occurring after discharge may have been missed.

Study outcomes

The primary outcome was the occurrence of at least one postoperative complication during the index hospitalization. Individual postoperative complications included surgical-site infection, anastomotic leakage, respiratory complications, renal complications, cardiac complications, and other clinically relevant complications.

The severity of postoperative complications was graded according to the Clavien-Dindo classification. For the primary severity analysis, complications were categorized as Clavien-Dindo grade I-II and grade ≥III, with grade ≥III considered major postoperative morbidity. When a patient experienced multiple complications, the highest Clavien-Dindo grade was used to classify overall complication severity.

The association between preoperative CRP and the occurrence and severity of postoperative complications was evaluated.

Statistical analysis

Data were analyzed using IBM SPSS Statistics for Windows, version 27.0 (IBM Corp., Armonk, USA). Continuous variables were assessed for distribution and summarized as means ± standard deviations (SDs) when approximately normally distributed and as medians with interquartile ranges (IQRs) when non-normally distributed. Categorical variables were presented as frequencies and percentages.

CRP was analyzed both as a continuous variable and as a categorical variable using the predefined threshold of >5 mg/L. Continuous CRP concentrations were compared between outcome groups using the independent-samples t test or Mann-Whitney U test, as appropriate. Associations between categorical variables were assessed using Pearson's chi-square test when expected cell counts were adequate. Fisher's exact test was used when expected frequencies were small or when the assumptions of the chi-square test were not satisfied.

For analyses involving individual postoperative complications, effect sizes were reported as odds ratios (ORs) with corresponding 95% confidence intervals (CIs) where estimable. When an OR could not be reliably estimated because of zero cells or sparse data, Fisher's exact test was reported instead. No multivariable regression analysis was performed because of the small sample size and limited number of major postoperative events. Therefore, all reported associations were unadjusted and should not be interpreted as demonstrating an independent predictive effect of CRP.

No formal adjustment for multiple comparisons was performed. Because multiple exploratory comparisons were conducted, particularly for individual postoperative complications, statistically significant findings were interpreted cautiously and considered hypothesis-generating. A two-sided P-value <0.05 was considered statistically significant.

Ethical considerations

The study was approved by the Institutional Review Board of Bangladesh Medical University, Dhaka, Bangladesh (approval number: BMU/2024/6490). The study was conducted in accordance with the ethical principles of the Declaration of Helsinki. Written informed consent was obtained from all participants before enrollment. Patient confidentiality was maintained throughout the study, and data were analyzed anonymously. Participation was voluntary, and participants were informed of their right to withdraw from the study at any time without affecting their clinical care. No financial incentive was provided to participants.

## Results

A total of 64 patients undergoing elective colorectal cancer surgery were included in the analysis. The mean age was 46.6 ± 15.3 years, with the largest proportion of patients aged 31-50 years (29; 45.3%). Forty-two patients (65.6%) were male, and 22 (34.4%) were female, giving a male-to-female ratio of 1.9:1. Most patients had a normal BMI, with 48 (75.0%) having a BMI of 18.5-24.9 kg/m². The mean BMI was 23.1 ± 3.0 kg/m². Thirty-four patients (53.1%) were smokers, while two (3.1%) reported alcohol consumption (Table 1).

**Table 1:** Demographic and clinical characteristics of the study population (n=64) Values are presented as n (%) unless otherwise specified. Continuous variables are presented as means ± standard deviations.

Characteristic	n (%)
Age, years	
≤30	11 (17.2)
31–50	29 (45.3)
51–70	22 (34.4)
>70	2 (3.1)
Mean ± SD	46.6 ± 15.3
Sex	
Male	42 (65.6)
Female	22 (34.4)
BMI, kg/m²	
<18.5	1 (1.6)
18.5–24.9	48 (75.0)
25.0–29.9	9 (14.1)
≥30.0	6 (9.4)
Mean ± SD	23.1 ± 3.0
Smoking status	
Smoker	34 (53.1)
Non-smoker	30 (46.9)
Alcohol consumption	
Yes	2 (3.1)
No	62 (96.9)

Most patients had stage III disease (59; 92.2%), while stage II disease was present in 4 (6.3%) patients and stage IV disease in 1 (1.6%) patient. Histological grade II was predominant (55; 85.9%), followed by grade III (6; 9.4%) and grade I (3; 4.7%). The most common tumor location was the ascending colon (22; 34.4%), followed by the cecum (12; 18.8%) and sigmoid colon (10; 15.6%). Laparoscopic surgery was performed in 36 (56.3%) patients, whereas 28 (43.7%) underwent open surgery. Adenocarcinoma was the predominant histological diagnosis (59; 92.2%), followed by mucinous adenocarcinoma (4; 6.3%) and leiomyosarcoma (1; 1.6%).

The mean hemoglobin concentration was 11.8 ± 1.1 g/dL. The mean WBC count was 11,050.2 ± 5,164.3/µL, and the mean platelet count was 234,734.4 ± 87,285.9/µL. The mean ESR was 31.8 ± 14.2 mm/h, while the mean preoperative CRP concentration was 5.9 ± 4.2 mg/L. Forty patients (62.5%) had a CRP concentration ≤5 mg/L, whereas 24 (37.5%) had CRP >5 mg/L. Five patients (7.8%) had hemoglobin <10 g/dL, 13 (20.3%) had a WBC count of >11,000/µL, 4 (6.3%) had a platelet count of <150,000/µL, and 20 (31.3%) had an ESR >35 mm/h.

According to the Clavien-Dindo classification, postoperative complications occurred in 43 (67.2%) of the 64 patients, while 21 (32.8%) had no postoperative complications. Among the 43 patients with complications, Grade I complications occurred in 20 (31.3%), Grade II in 13 (20.3%), Grade III in 6 (9.4%), Grade IV in 2 (3.1%), and Grade V in 2 (3.1%) patients (Table 2). The most frequently documented specific postoperative complication was wound infection, occurring in 17 (26.6%) patients, followed by anastomotic leakage in 6 (9.4%), respiratory complications in 6 (9.4%), and renal complications in 1 (1.6%). Two postoperative deaths (3.1%) occurred during the study period; one was associated with suspected venous thromboembolism, while the other occurred following postoperative myocardial infarction.

**Table 2:** Postoperative complications according to the Clavien-Dindo classification (n=64) Data are presented as n (%). Grade 0 indicates no postoperative complications. Grades I-V represent increasing severity of postoperative complications according to the Clavien-Dindo classification. Percentages are calculated using the total study population (n=64).

Clavien-Dindo classification	n (%)
No postoperative complication (Grade 0)	21 (32.8)
Grade I	20 (31.3)
Grade II	13 (20.3)
Grade III	6 (9.4)
Grade IV	2 (3.1)
Grade V	2 (3.1)
Total	64 (100.0)

Among the 43 patients who developed postoperative complications, 10 (23.3%) had severe complications (Clavien-Dindo grade ≥III), while 33 (76.7%) had grade I-II complications. There was no statistically significant association between complication severity and age, sex, BMI, smoking status, alcohol consumption, hemoglobin level, white blood cell count, or platelet count (all P>0.05). Severe complications were more frequent among patients with BMI ≥25.0 kg/m² than among those with BMI <25.0 kg/m² (60.0% vs. 27.3%), although the difference did not reach statistical significance (P=0.073). Similarly, an ESR >35 mm/h was more common among patients with grade ≥III complications than among those with grade I-II complications (70.0% vs. 36.4%), but this association was not statistically significant (P=0.079) (Table 3).

**Table 3:** Association of demographic and preoperative laboratory characteristics with Clavien-Dindo complication severity among patients with postoperative complications (n=43) Data are presented as n (%). Clavien-Dindo grade ≥III represented severe postoperative complications, whereas grade <III included grades I and II. Categorical variables were compared using Pearson’s chi-square test or Fisher’s exact test, as appropriate. Degrees of freedom (df) are reported for Pearson’s chi-square tests; Fisher’s exact test does not require reporting of degrees of freedom. A two-sided P-value <0.05 was considered statistically significant. BMI, body mass index; WBC, white blood cell; ESR, erythrocyte sedimentation rate; CRP, C-reactive protein

Variable	Clavien-Dindo grade ≥III (n=10), n (%)	Clavien-Dindo grade I-II (n=33), n (%)	Test	P-value
Age (years)				
>50	4 (40.0)	18 (54.5)	Fisher’s exact test	0.488
≤50	6 (60.0)	15 (45.5)		
Sex				
Male	7 (70.0)	24 (72.7)	Fisher’s exact test	1.000
Female	3 (30.0)	9 (27.3)		
BMI (kg/m²)				
≥25.0	6 (60.0)	9 (27.3)	Fisher’s exact test	0.073
<25.0	4 (40.0)	24 (72.7)		
Smoking status				
Yes	6 (60.0)	18 (54.5)	Fisher’s exact test	1.000
No	4 (40.0)	15 (45.5)		
Alcohol consumption				
Yes	0 (0.0)	2 (6.1)	Fisher’s exact test	1.000
No	10 (100.0)	31 (93.9)		
Hemoglobin (g/dL)				
<10.0	2 (20.0)	3 (9.1)	Fisher’s exact test	0.575
≥10.0	8 (80.0)	30 (90.9)		
WBC count (/µL)				
>11,000	4 (40.0)	8 (24.2)	Fisher’s exact test	0.427
≤11,000	6 (60.0)	25 (75.8)		
Platelet count (/µL)				
<150,000	2 (20.0)	2 (6.1)	Fisher’s exact test	0.226
≥150,000	8 (80.0)	31 (93.9)		
ESR (mm/h)				
>35	7 (70.0)	12 (36.4)	Fisher’s exact test	0.079
≤35	3 (30.0)	21 (63.6)		
CRP (mg/L)				
>5	10 (100.0)	12 (36.4)	Fisher’s exact test	<0.001
≤5	0 (0.0)	21 (63.6)		

In contrast, preoperative CRP showed a statistically significant association with complication severity. All 10 patients with Clavien-Dindo grade ≥III complications had a CRP level of >5 mg/L, compared with 12 of 33 patients (36.4%) with grade I-II complications (P<0.001). Conversely, none of the patients with severe complications had a CRP level ≤5 mg/L, whereas 21 (63.6%) of those with grade I-II complications had CRP ≤5 mg/L. These findings indicate that elevated preoperative CRP was significantly associated with more severe postoperative complications in this cohort (Table 3).

Among the preoperative inflammatory markers evaluated, elevated CRP showed the most consistent association with postoperative complications. Patients with CRP >5 mg/L had a substantially higher frequency of wound infection than those with CRP ≤5 mg/L (58.3% vs. 7.5%; Fisher’s exact test, P<0.001). Similarly, respiratory complications occurred more frequently in patients with elevated CRP (20.8% vs. 2.5%; Fisher’s exact test, P=0.024). Anastomotic leakage was also significantly more common among patients with CRP >5 mg/L than among those with CRP ≤5 mg/L (25.0% vs. 0.0%; Fisher’s exact test, P=0.002). In contrast, renal complications were uncommon and did not differ significantly according to CRP status (4.2% vs. 0.0%; P=0.375) (Table 4).

**Table 4:** Association between demographic and preoperative laboratory characteristics and specific postoperative complications among patients with colorectal cancer (n=64) Data are presented as n (%). P-values were calculated using the two-sided Fisher’s exact test for comparisons between categorical variables. Statistical significance was defined as P<0.05. BMI, body mass index; WBC, white blood cell; ESR, erythrocyte sedimentation rate; CRP, C-reactive protein

Variable	Wound infection, n (%)	Respiratory complication, n (%)	Renal complication, n (%)	Anastomotic leak, n (%)
Age (years)				
>50 (n=24)	9 (37.5)	5 (20.8)	1 (4.2)	4 (16.7)
≤50 (n=40)	8 (20.0)	1 (2.5)	0 (0.0)	2 (5.0)
Test; P-value	Fisher's; P=0.151	Fisher's; P=0.024	Fisher's; P=0.375	Fisher's; P=0.186
Sex				
Male (n=42)	13 (31.0)	4 (9.5)	1 (2.4)	5 (11.9)
Female (n=22)	4 (18.2)	2 (9.1)	0 (0.0)	1 (4.5)
Test; P-value	Fisher's; P=0.375	Fisher's; P=1.000	Fisher's; P=1.000	Fisher's; P=0.655
BMI (kg/m²)				
≥25.0 (n=15)	7 (46.7)	5 (33.3)	1 (6.7)	3 (20.0)
<25.0 (n=49)	10 (20.4)	1 (2.0)	0 (0.0)	3 (6.1)
Test; P-value	Fisher's; P=0.091	Fisher's; P=0.002	Fisher's; P=0.234	Fisher's; P=0.135
Smoking status				
Yes (n=34)	11 (32.4)	3 (8.8)	1 (2.9)	5 (14.7)
No (n=30)	6 (20.0)	3 (10.0)	0 (0.0)	1 (3.3)
Test; P-value	Fisher's; P=0.396	Fisher's; P=1.000	Fisher's; P=1.000	Fisher's; P=0.202
Hemoglobin (g/dL)				
<10.0 (n=5)	3 (60.0)	4 (80.0)	1 (20.0)	2 (40.0)
≥10.0 (n=59)	14 (23.7)	2 (3.4)	0 (0.0)	4 (6.8)
Test; P-value	Fisher's; P=0.112	Fisher's; P<0.001	Fisher's; P=0.078	Fisher's; P=0.065
WBC count (/µL)				
>11,000 (n=13)	6 (46.2)	5 (38.5)	1 (7.7)	3 (23.1)
≤11,000 (n=51)	11 (21.6)	1 (2.0)	0 (0.0)	3 (5.9)
Test; P-value	Fisher's; P=0.089	Fisher's; P<0.001	Fisher's; P=0.203	Fisher's; P=0.092
Platelet count (/µL)				
<150,000 (n=4)	2 (50.0)	4 (100.0)	1 (25.0)	1 (25.0)
≥150,000 (n=60)	15 (25.0)	2 (3.3)	0 (0.0)	5 (8.3)
Test; P-value	Fisher's; P=0.285	Fisher's; P<0.001	Fisher's; P=0.063	Fisher's; P=0.332
ESR (mm/h)				
>35	9 (45.0)	3 (15.0)	1 (5.0)	4 (20.0)
≤35	8 (18.2)	3 (6.8)	0 (0.0)	2 (4.5)
Test; P-value	Fisher's; P=0.034	Fisher's; P=0.366	Fisher's; P=0.313	Fisher's; P=0.071
CRP (mg/L)				
>5	14 (58.3)	5 (20.8)	1 (4.2)	6 (25.0)
≤5	3 (7.5)	1 (2.5)	0 (0.0)	0 (0.0)
Test; P-value	Fisher's; P<0.001	Fisher's; P=0.024	Fisher's; P=0.375	Fisher's; P=0.002

Other preoperative variables showed less consistent associations with postoperative complications. Elevated ESR (>35 mm/h) was significantly associated with wound infection (45.0% vs. 18.2%; P=0.034), while age >50 years, BMI ≥25.0 kg/m², hemoglobin <10.0 g/dL, WBC count >11,000/µL, and platelet count <150,000/µL were significantly associated with respiratory complications. However, the particularly strong and statistically significant associations between CRP elevation and wound infection, respiratory complications, and anastomotic leakage suggest that preoperative CRP may be an important marker of postoperative morbidity in this cohort (Table 4).

## Discussion

The present prospective observational study evaluated the association between preoperative CRP and postoperative complications among patients undergoing elective colorectal cancer surgery. The principal finding was that elevated preoperative CRP was significantly associated with several measures of postoperative morbidity. Patients with CRP >5 mg/L had significantly higher rates of wound infection, respiratory complications, and anastomotic leakage and were more likely to experience higher grade postoperative complications. These findings suggest that preoperative CRP may provide useful information regarding postoperative risk in patients undergoing colorectal cancer surgery.

In this cohort, elevated preoperative CRP may have served as a marker of an underlying inflammatory burden present before surgery. The association with several postoperative outcomes is biologically plausible because patients entering major abdominal surgery with greater systemic inflammatory activity may have low physiological reserve [12]. Nevertheless, the observational design does not allow determination of whether elevated CRP itself contributes to these complications or simply reflects other clinical or tumor-related characteristics.

The study population had a mean age of 46.6 ± 15.3 years, and 65.6% of patients were male. Stage III disease predominated, accounting for 59 (92.2%) patients, while adenocarcinoma was the most frequent histological diagnosis. The relatively young age distribution and predominance of advanced-stage disease may reflect local referral patterns, delayed presentation, and differences in cancer detection practices. These characteristics are important when interpreting the inflammatory profile and postoperative outcomes observed in this cohort.

The mean preoperative CRP concentration was 5.9 ± 4.2 mg/L, with 24 (37.5%) patients having CRP >5 mg/L. Previous studies have reported associations between systemic inflammation and adverse postoperative outcomes following colorectal cancer surgery [13,14]. Moyes et al. demonstrated an association between preoperative systemic inflammation and postoperative infectious complications following potentially curative colorectal cancer surgery [15]. The present findings are consistent with this observation and further demonstrate associations between elevated CRP and several specific postoperative complications.

The strongest association between CRP and an individual complication was observed for wound infection. Wound infection occurred in 14 (58.3%) patients with CRP >5 mg/L compared with 3 (7.5%) patients with CRP ≤5 mg/L (P<0.001). This marked difference suggests that an elevated preoperative inflammatory state may identify patients who are more susceptible to postoperative surgical-site infection or who have reduced physiological reserve. However, CRP is nonspecific and may also reflect other unmeasured clinical, infectious, or tumor-related factors [16]. Therefore, the association should not be interpreted as evidence that CRP directly causes wound infection.

Elevated CRP was also significantly associated with respiratory complications, which occurred in 5 (20.8%) patients with CRP >5 mg/L compared with 1 (2.5%) patient with CRP ≤5 mg/L (P=0.025). This finding is compatible with the broader relationship between systemic inflammatory status and postoperative morbidity following major colorectal surgery [17]. Anastomotic leakage was likewise more frequent among patients with elevated CRP, occurring in 6 (25.0%) patients with CRP >5 mg/L compared with none among those with CRP ≤5 mg/L (P=0.002). Although this association is clinically important, the small number of leakage events warrants cautious interpretation. No significant association was observed between CRP and renal complications (P=0.375), although only one renal complication occurred in the cohort.

CRP was also associated with the severity of postoperative complications. Among the 43 patients who developed postoperative complications, 10 (23.3%) experienced Clavien-Dindo grade III or higher complications. All 10 patients with severe complications had preoperative CRP >5 mg/L, compared with 12 of 33 patients (36.4%) with grade I or II complications (P<0.001). This finding indicates a strong association between elevated preoperative CRP and more severe postoperative morbidity. Nevertheless, the relatively small number of severe events limits the precision of this estimate, and confirmation in larger cohorts is necessary.

The potential practical relevance of CRP is particularly important in resource-constrained settings. CRP testing is relatively inexpensive, routinely available, and easy to incorporate into preoperative assessment [18]. If validated in larger studies, preoperative CRP could complement routine clinical evaluation by identifying patients who may require closer perioperative surveillance or additional optimization. However, CRP should not be considered a standalone determinant of surgical management because it lacks specificity and must be interpreted alongside clinical, nutritional, oncological, and operative factors [19,20].

Several limitations should be considered when interpreting these findings. First, the sample size was relatively small (n=64), with limited numbers of individual postoperative complications and severe events. This reduced the precision of estimates, particularly for less frequent outcomes such as anastomotic leakage, renal complications, and Clavien-Dindo grade III or higher complications. Second, the study was conducted at a single center using purposive sampling, which may introduce selection bias and limit the generalizability of the findings to other institutions and patient populations.

Third, the analyses were based primarily on unadjusted associations. Therefore, potential confounding by age, comorbidities, nutritional status, tumor characteristics, disease stage, type of surgical procedure, and other perioperative factors cannot be excluded. The observed associations should consequently not be interpreted as evidence that elevated CRP is an independent predictor of postoperative complications. Fourth, CRP is a nonspecific inflammatory biomarker and may be elevated because of infection, chronic inflammatory conditions, tumor-related inflammation, or other clinical factors unrelated to the surgical procedure. Although CRP was measured preoperatively, the study could not establish the specific cause of an elevated CRP level in individual patients. Fifth, postoperative complications occurring after discharge may have been under-ascertained if patients sought treatment at other healthcare facilities or were unavailable for follow-up. Sixth, the use of a CRP threshold of >5 mg/L was based on the predefined analytical approach and should not be considered an established clinical threshold for risk stratification in colorectal cancer surgery. Finally, although the prospective design allowed systematic assessment of preoperative CRP and postoperative outcomes, the observational nature of the study prevents determination of a causal relationship between elevated CRP and postoperative complications.

Overall, elevated preoperative CRP was significantly associated with postoperative morbidity, including wound infection, respiratory complications, anastomotic leakage, and greater complication severity. These findings support the potential role of preoperative CRP as a simple adjunctive marker of perioperative risk in patients undergoing colorectal cancer surgery. However, the observed associations should be interpreted cautiously because of the small sample size, single-center design, purposive sampling, and potential for residual confounding. Larger multicenter prospective studies with adequate numbers of postoperative events and appropriate adjustment for relevant clinical and oncological factors are required to determine the independent prognostic value and clinical utility of preoperative CRP.

## Conclusions

A preoperative CRP level of >5 mg/L was significantly associated with postoperative morbidity, including wound infection, respiratory complications, anastomotic leakage, and higher Clavien-Dindo complication severity among patients undergoing elective colorectal cancer surgery. Elevated CRP may therefore have potential value as an adjunctive marker of perioperative risk assessment. However, these findings represent unadjusted associations and should not be interpreted as evidence of an independent or causal effect of CRP. The small sample size, limited number of postoperative events, single-center design, purposive sampling, and potential for residual confounding further limit the strength and generalizability of the findings. Larger multicenter prospective studies with appropriate adjustment for relevant clinical and oncological factors are needed to validate the clinical utility of preoperative CRP in colorectal cancer surgery.
